# Tumor genetic heterogeneity analysis of chronic sun‐damaged melanoma

**DOI:** 10.1111/pcmr.12851

**Published:** 2019-12-23

**Authors:** Adriana Sanna, Katja Harbst, Iva Johansson, Gustav Christensen, Martin Lauss, Shamik Mitra, Frida Rosengren, Jari Häkkinen, Johan Vallon‐Christersson, Håkan Olsson, Åsa Ingvar, Karolin Isaksson, Christian Ingvar, Kari Nielsen, Göran Jönsson

**Affiliations:** ^1^ Department of Clinical Sciences Lund Division of Oncology and Pathology Lund University Lund Sweden; ^2^ Department of Clinical Pathology Skåne University Hospital Lund Sweden; ^3^ Department of Dermatology Skåne University Hospital Lund Sweden; ^4^ Department of Clinical Sciences Lund Division of Dermatology and Venereology Lund University Lund Sweden; ^5^ Department of Clinical Sciences Lund Division of Surgery Skåne University Hospital Lund University Lund Sweden; ^6^ Department of Dermatology Nordvästra Skåne Teaching Hospital Lund Sweden

**Keywords:** chronic sun damage, heterogeneity, in situ, invasive, melanoma

## Abstract

Chronic sun‐damaged (CSD) melanoma represents 10%–20% of cutaneous melanomas and is characterized by infrequent *BRAF* V600E mutations and high mutational load. However, the order of genetic events or the extent of intra‐tumor heterogeneity (ITH) in CSD^high^ melanoma is still unknown. Ultra‐deep targeted sequencing of 40 cancer‐associated genes was performed in 72 in situ or invasive CMM, including 23 CSD^high^ cases. In addition, we performed whole exome and RNA sequencing on multiple regions of primary tumor and multiple in‐transit metastases from one CSD^high^ melanoma patient. We found no significant difference in mutation frequency in melanoma‐related genes or in mutational load between in situ and invasive CSD^high^ lesions, while this difference was observed in CSD^low^ lesions. In addition, increased frequency of *BRAF* V600K, *NF1,* and *TP53* mutations (*p* < .01, Fisher's exact test) was found in CSD^high^ melanomas. Sequencing of multiple specimens from one CSD^high^ patient revealed strikingly limited ITH with >95% shared mutations. Our results provide evidence that CSD^high^ and CSD^low^ melanomas are distinct molecular entities that progress via different genetic routes.

**Significance**

Increased genetic understanding of the transition from in situ to invasive melanoma is fundamental for proper diagnosis and to understand how melanoma develops. In this study, we found no mutational difference between in situ and invasive lesions from patients with chronic sun‐damaged melanoma (CSD^high^). We further demonstrated that intra‐tumor heterogeneity is limited throughout progression in a patient case with CSD^high^ melanoma. Overall, we conclude different degree of genetic heterogeneity in CSD^high^ and CSD^low^ melanoma.

## 1 INTRODUCTION

Melanoma can broadly be categorized according to its origin, that is, whether it is localized on skin that shows high chronic sun damage (CSD) or not. CSD^high^ and CSD^low^ melanomas differ in many aspects, such as anatomic site of the primary tumor and patient age (Anderson, Pfeiffer, Tucker, & Rosenberg, 2009; Yeh et al., 2016). While CSD^high^ melanomas are frequently located on the head and neck and dorsal surfaces of extremities, CSD^low^ melanomas are mainly located on intermittently sun exposed parts of the body (Yeh et al., 2016). The main CSD^high^ histological types are lentigo maligna melanoma (LMM), CSD^high^ nodular melanoma, and desmoplastic melanoma (DM). DM is rare (approximately less than 5% of all melanoma cases) and can arise in association with LMM. Lentigo maligna (LM), the in situ phase of LMM, can easily be overlooked, both by patients and during medical examinations, due to its slow growth and strong resemblance to benign hyperpigmented skin lesions. Interestingly, only about 5% of LM gain vertical growth phase properties and transform into invasive LMM (Weinstock & Sober, 1987). Although there are important biological differences (Yeh et al., 2016), clinical management and prognosis of CSD^high^ melanoma does not differ from that of other CMM (Abdelmalek, Loosemore, Hurt, & Hruza, 2012; Koh et al., 1984). However, a recent study indicates that CSD^high^ tumors express increased levels of PD‐L1 and therefore may respond to PD‐1 inhibition (Kaunitz et al., 2017). Indeed, high response rate was observed in a clinical trial investigating efficacy of PD‐1 blockade in DM, which may partly be attributed to higher mutational burden in CSD^high^ compared to CSD^low^ melanomas (Eroglu et al., 2018).

Knowledge of the mutational landscape of CSD^high^ melanomas is limited. They rarely harbor *BRAF* V600E mutation, but have recurrent *NFKBIE* promoter mutations and *KIT* aberrations, and increased mutational load (Boussemart et al., 2018; Curtin, Busam, Pinkel, & Bastian, 2006; Curtin et al., 2005; Eroglu et al., 2018; Shain, Garrido, et al., 2015). The genetic landscape of in situ CSD^high^ lesions is largely unknown (Yeh et al., 2016). Hence, we examined the mutational patterns of in situ and invasive CMM, including CSD^high^ melanomas. Additionally, in order to resolve ITH in CSD^high^ melanoma, we performed genomic analysis of multiple biopsies from one CSD^high^ melanoma patient. These data unveiled striking similarity of all specimens on the different genomic levels, with a few notable differences.

## 2 METHODS

### 2.1 Patient cohort

All patients included in this study were part of BioMEL, a prospective study in tertiary dermatological, surgical, and oncological departments in teaching and university hospitals in the south of Sweden. BioMEL is aiming at improving risk prediction, diagnosis, prognosis, and treatment response by means of accruing clinical information and a biobank of early stage melanoma lesions and other cutaneous lesions that resemble melanoma. We defined CSD^high^ melanoma as lesions diagnosed at 55 years of age or older, on the head and neck/shoulder region or dorsal surfaces of hands and feet. CSD^low^ melanomas include other skin melanomas (Yeh et al., 2016). One patient presented with an ulcerated 25 mm diameter primary melanoma of unclassified histopathological subtype with spindle‐like cell morphology, Breslow 16 mm, Clark V, no signs of regression and concurrent multiple satellite, and in‐transit metastases. This melanoma was located on the shoulder. From this patient, 5 primary tumor fragments (PT) and 7 in‐transit metastases (IT) were surgically removed and immediately stored at −80°C. Normal skin adjacent to the primary tumor was used as matched normal control. The patient had not received any therapy prior to surgery. Informed written consent was obtained from all participants. The study was approved by the Regional Ethical Committee (Dnr. 101/2013).

### 2.2 Analytical procedures

#### 2.2.1 Tissue collection

Dermatoscopy‐guided full skin tumor biopsies (1mm in diameter) were collected by trained dermatologists from the suspected melanoma within 30 s after primary surgery of the lesion, thereafter snap frozen and stored at −80°C. Included investigators who were involved in taking the tumor biopsies of primary melanocytic tumors were all specialized in diagnosing pigmented lesions with dermoscopy. All melanocytic tumors were observed and preoperatively evaluated according to common dermatoscopic algorithms, preferably pattern analysis, or the 7‐point checklist algorithm (Argenziano et al., 1998). The majority of primary tumors were photographed both macroscopically and dermatoscopically before surgery. The clinical examination (including palpation) and the dermatoscopic view guided the involved investigator to where the suspected melanoma might possibly be thickest or most “aggressive”‐looking (Argenziano, Fabbrocini, Carli, De Giorgi, & Delfino, 1999; Carli, de Giorgi, Palli, Giannotti, & Giannotti, 2000; Stante, De Giorgi, Cappugi, Giannotti, & Carli, 2001). Hence, the investigators tried to predict which part of the tumor that would be most valuable to the pathologist to preserve intact, without the possible interference of a biopsy taken in that specific area. Therefore, the involved investigators were instructed to take the biopsies in close vicinity of the presumed thickest or most aggressive‐looking area of the melanocytic tumors.

#### 2.2.2 Nucleic acid extraction and sequencing

From the biopsies, tissue of interest (in situ or invasive parts in epidermis/dermis) was separated from adjacent subcutaneous fat under supervision of an experienced dermatopathologist (IJ). DNA and RNA were extracted from the tissue using AllPrep DNA/RNA Mini Kit (Qiagen).

#### 2.2.3 Targeted gene sequencing

Ultra‐deep sequencing of selected genes (Table S1) was performed using TruSeq Custom Amplicon Low Input workflow and NextSeq500 (Illumina) on all tissue of interest samples. Mean coverage of 5,758× was achieved (mean coverage *per* sample, range 838×–12,958×). PT1 and IT3 were excluded from the dataset due to low mutant allele frequency.

#### 2.2.4 Whole exome sequencing

Tumor and matched normal DNA samples from the CSD^high^ case (*n* = 13) were subjected to library preparation as described previously (Lauss et al., 2017). Libraries were sequenced on a HiSeq 2500 or NextSeq. Median target coverage for the libraries ranged from 68× to 126×.

#### 2.2.5 RNA sequencing

RNA‐seq was performed on all samples (tumor and non‐tumor) from the CSD^high^ case as described previously (Harbst et al., 2016). Details of WES and RNA‐seq data analysis are outlined in the Appendix S1 (also see Figure S1). Processed gene expression dataset is available at GEO under the accession number GSE139362.

#### 2.2.6 Validation of mutation related findings

For validation of mutation frequencies in CSD subtypes, an independent dataset was used (Cirenajwis et al., 2017). The dataset represented combined mutational and clinical data from four independent studies comprising 870 melanoma tumors. From this dataset, 479 CMM cases (76 primary tumors and 399 metastases) were included into the analysis. CSD^high^ and CSD^low^ cases were defined as in the study cohort (see above), yielding 444 non‐CSD (67 primary tumors and 377 metastasis), and 35 CSD^high^ (9 primary tumors and 26 metastases) cases. Mutations were derived from 1,461 genes; *TERT* promoter was not part of the target design.

#### 2.2.7 Statistical Analysis

All statistical tests were two‐sided and performed in *R*, and a *p*‐value of <.05 was considered statistically significant. The specific tests are indicated in the main text or figure legend.

## 3 RESULTS

### 3.1 Ultra‐deep sequencing of invasive and in situ melanoma lesions

In this study, 184 patients were enrolled at the dermatology clinics at two sites in southern Sweden (Lund and Helsingborg) when there was suspicion of melanoma or melanoma in situ. After histopathological diagnosis, 72 were identified as in situ or invasive primary CMM, representing the cohort of this study. Tumors were further categorized as either CSD^high^ or CSD^low^ (Table 1), according to anatomic site and age at diagnosis (Yeh et al., 2016), as described in Methods. To determine mutations in melanoma‐related genes (Table S1), we applied targeted ultra‐deep sequencing of 40 melanoma relevant genes to the tumor samples and obtained an average coverage of 5,758×.

**Table 1:** Clinical features of the melanoma cohort recruited in Helsingborg (*n* = 32) and Lund (*n* = 41)

	Entire cohort (*n* = 72)	In situ CSD^low^ (*n* = 20)	Invasive CSD^low^ (*n* = 29)	In situ CSD^high^ (*n* = 12)	Invasive CSD^high^ (*n* = 11)	*p*‐value
Patient characteristics						
Gender *n* (%)						
Female	29 (40)	8 (40)	13 (45)	4 (33)	4 (36)	ns
Male	43 (60)	12 (60)	16 (55)	8 (67)	7 (64)	ns
Age at diagnosis median (range)	68 (17–93)	65 (17–89)	58 (37–89)	76 (62–86)	77 (61–93)	.0037
Tumor characteristics						
Breslow thickness						
mm (range)	0.75 (0.3–16)	NA	0.7 (0.3–12)	NA	1.1 (0.34–16)	ns

	SSM	NM	LM	LMM
Tumor subtypes				
Invasive CSD^low^	25	4	0	1
In situ CSD^high^	1	0	7	0
Invasive CSD^high^	7	0	0	4

Abbreviations: LM, lentigo maligna; LMM, lentigo maligna melanoma; SSM, superficial spreading melanoma; NM, nodular melanoma.

### 3.2 Oncogenic mutations in CSD^high^ and CSD^low^ cutaneous melanomas

**Figure 1:**
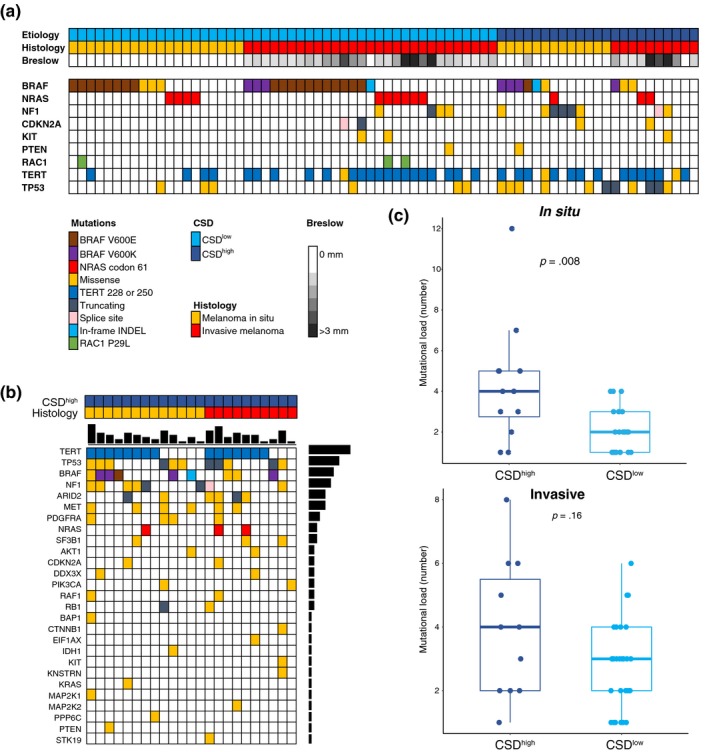
Mutations detected by targeted ultra‐deep sequencing in the cutaneous melanoma cohort. (a) Mutations in the major melanoma or cancer genes. Lesions are ordered according to CSD subtype. (b) All mutations from the targeted sequencing analysis identified in the CSD^high^ samples. (c) Mutational load of the melanoma subtypes from the targeted sequencing according to in situ or invasive (top panel in situ, bottom panel invasive). Y‐axis indicates the number of detected mutations. *p‐*value was calculated using Wilcoxon signed‐rank test

Although the mutational landscape of CMM has been thoroughly described (Berger et al., 2012; Cancer Genome Atlas, 2015; Cirenajwis et al., 2017; Hayward et al., 2017; Hodis et al., 2012; Krauthammer et al., 2012), the majority of studies thus far have been conducted on CSD^low^ metastatic CMM. In this study, we found frequent mutations in *BRAF* (*n* = 35, 49%); the majority were V600E (*n* = 20, 57%) with less frequent substitutions leading to V600K (*n* = 7, 20%), K601E (*n* = 4, 11%), and complex hotspot mutations (T599dup and V600_K601delinsE). Although *BRAF* mutations were equally frequent between the groups (39% in CSD^high^ and 53% in CSD^low^, *p* = .45), within *BRAF* hotspot mutations, the proportion of V600K mutations was higher in CSD^high^ than in CSD^low^ cases (*p* = .009, Fisher's exact test, Figure 1a–b), supporting previous studies (Menzies et al., 2012; Stadelmeyer et al., 2014). All *NRAS* mutations (*n* = 13, 18%) were mapped to the Q61 codon and were mutually exclusive to *BRAF* mutations. Mutations in *NF1* (*n* = 12, 17%) and *TP53* (*n* = 17, 24%) were more frequent in CSD^high^ as compared to CSD^low^ melanoma (*NF1*: 35% vs. 8%, *p* = .007; *TP53*: 48% vs. 12%, *p* = .002, Fisher's exact test). Moreover, *TERT* promoter hotspot mutations were frequent in all histopathological types, and in CSD^low^ lesions, they were more frequent among the invasive than the in situ lesions (*p* = .002, Fisher's exact test). Three cases harbored *KIT* mutations: two invasive CSD^low^ SSMs (V474A and T666L) and one CSD^high^ CMM (L576P). The first two mutations have not been identified in COSMIC, suggesting a passenger role, while the latter has been detected in 124 independent samples and predicted pathogenic, indicating a driver role (COSMIC accession date April 18, 2019). Finally, we found three cases with hotspot *RAC1* mutations affecting Proline 29 in co‐occurrence with *BRAF* or *NRAS* hotspot mutations. The allelic frequencies of these key melanoma drivers are shown in Figure S2.

Further highlighting chronic UV exposure as a major driver of CSD^high^ tumor initiation, we observed a significant increase in mutational load in CSD^high^ compared to CSD^low^ lesions (*p* = .0048, Wilcoxon signed‐rank test, Figure S3a). This effect was more pronounced in the in situ lesions (*p* = .008, Wilcoxon signed‐rank test, Figure 1c, top panel) than in the invasive lesions (*p* = .16, Wilcoxon signed‐rank test, Figure 1c, bottom panel). Importantly, there was no significant difference in mutation frequency in any of the melanoma driver genes nor in mutational load between the two stages in the CSD^high^ melanomas (*p* = .93, Wilcoxon signed‐rank test, Figure S3b, left panel), while there was a significant difference in mutational load in the CSD^low^ lesions (*p* = .05, Wilcoxon signed‐rank test, Figure S3b, right panel).

To validate our findings, we turned to external mutational dataset (Cirenajwis et al., 2017), from which 479 CMM (76 primary and 399 metastases) were included in the analysis (Table S2; Figure S4a). Supporting our results, CSD^high^ tumors had significantly higher mutational load (*p* < .001, Wilcoxon signed‐rank test, Figure S4b), increased frequency of *BRAF* V600K, *NF1,* and *TP53* (*p* = .0003, *p* = .02, and *p* = .003, respectively, Fisher's exact test) mutations and trend toward elevated frequency of *KIT* mutations (9% vs. 3%), as compared to CSD^low^ tumors.

### 3.3 Analysis of intra‐tumor transcriptional heterogeneity in CSD^high^ melanoma

**Figure 2:**
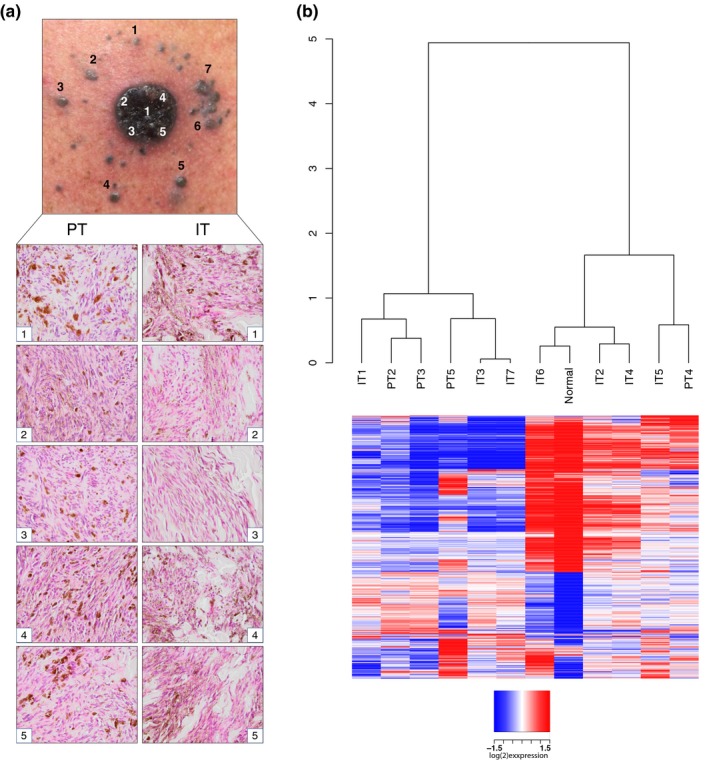
Intra‐tumor heterogeneity in CSD^high^ melanoma. (a) Tissue specimens were derived from the indicated primary tumor (PT) regions and satellite/in‐transit metastases (IT). Histological appearance of the samples by H&E staining is presented. (b) Dendrogram of unsupervised clustering of the PT and IT specimens based on the expression of 1,500 most varying genes (top panel) and heatmap of their expression within the specimens of the CSD^high^ intra‐tumor heterogeneity case (bottom panel)

**Figure 3:**
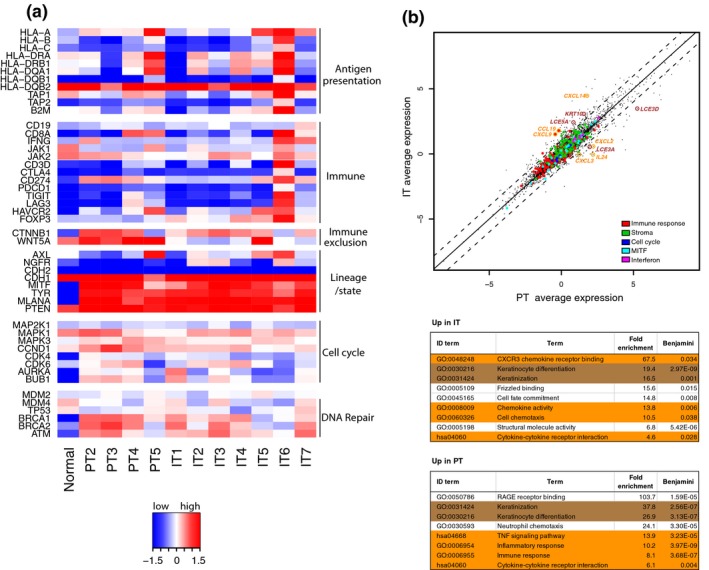
Transcriptional intra‐tumor heterogeneity in CSD^high^ melanoma. (a) Gene expression heatmap of selected genes. (b) Scatter plot of average gene expression of all PT versus all IT specimens. Highlighted in color are genes comprising biologically important modules as in the legend (Cirenajwis et al., 2017). Circles highlight selected genes with log_2_ fold change between average PT expression and average IT expression above 1 or below −1, for which GO term DAVID analysis found significant difference (orange = chemokine activity; brown = keratinocyte differentiation) (top panel), and significant (Benjamini corrected *p* < .05) terms in the GO term DAVID analysis (bottom panel)

To resolve ITH in CSD^high^ melanoma, we focused on one CSD^high^ case with a *KIT* mutation (Figure 1a). This patient presented clinically with a histologically unclassified primary melanoma (PT) with multiple satellite and in‐transit metastases (IT) on the right part of the head and neck region (Figure 2a). Histological examination showed pigmentation, solar elastosis, inflammation, and spindle‐like morphology of the melanoma cells (Figure 2a), typical features of CSD^high^ melanomas (Smoller, 2006). We performed ultra‐deep targeted sequencing, WES, and RNA‐seq of five regions from the primary tumor and seven synchronous IT. Unsupervised clustering of gene expression data revealed no differences between PT and IT specimens, with samples dividing into two main clusters by similarity to the normal skin sample (Figure 2b). Indeed, all specimens displayed similar expression of pigmentation, cell cycle, DNA repair, and immune programs (Figure 3a). PT5 and IT6 represented an exception since they displayed increased levels of antigen presentation and immune genes, respectively, probably due to higher immune cell infiltration. There was no difference in the expression of biologically important gene modules between PT and IT specimens (Figure 3b). In fact, genes upregulated in IT (log_2_ fold change > 1) or in PT (log_2_ fold change <−1) belonged to the same GO terms (Figure 3b), indicating transcriptional similarity of PT and IT samples. Finally, supervised analysis did not yield any gene with significantly different expression between these groups (Figure S5). In conclusion, all tumor specimens showed a high degree of similarity at the transcriptional level.

### 3.4 Intra‐tumor mutational heterogeneity in CSD^high^ melanoma

**Figure 4:**
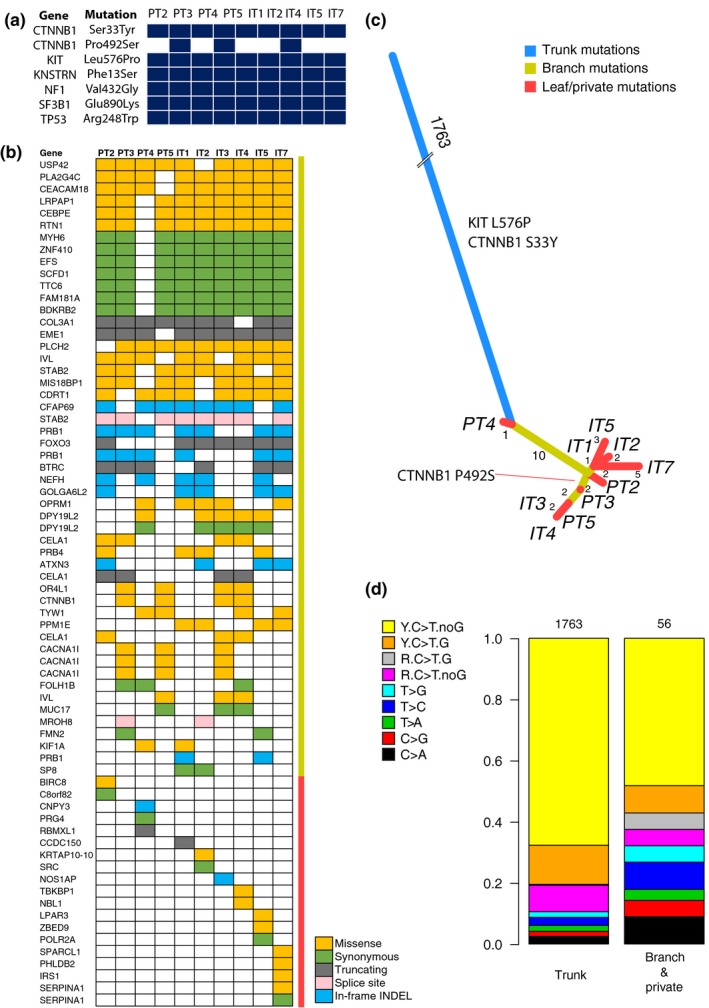
Mutational heterogeneity in the CSD^high^ melanoma patient. (a) Mutations detected by targeted sequencing of 40 melanoma genes. (b) Heatmap of branch and private mutations identified by WES in the tumor specimens from the patient. Color indicates a mutation, while white indicates its absence. Yellow and red bars next to the mutation heatmap mark branch and private mutations, respectively. (c) Phylogenetic tree representation of the WES mutations. The length of the branches is proportional to the number of somatic mutations; trunk has been shortened to 50 mutations. Trunk mutations are in blue; mutations shared by at least two regions are in yellow; leaf/private mutations for each region are in red. d, Mutation signatures in the WES data from the CSD^high^ melanoma patient, for trunk vs. branch and private mutations. C to T substitutions (C > T) are divided into four groups depending on the preceding purine (R) or pyrimidine (Y), and succeeding G vs. any other nucleotide

We then asked whether the similarity observed at the transcriptional level was also present at the genetic level. Examination of the ultra‐deep sequencing panel data from the analyzed regions revealed six mutations in cancer genes in all samples and one heterogeneous mutation (*CTNNB1* P492S) confined to PT3, PT5, and IT4 (Figure 4a). The high median sequence coverage at these mutation sites (13,000×) supported the true nature of this heterogeneous pattern.

Therefore, we performed WES on these specimens, including an adjacent skin sample as matched normal control, with average target coverage of 68–126×. We applied a rigorous mutation calling pipeline aimed at revealing the true mutational heterogeneity by minimizing the influence from technical parameters, *for example* variation in tumor cell content between samples (Appendix S1). PT1 and IT6 were excluded from further analysis due to low tumor purity. In total, we identified 1,844 somatically acquired mutations in all tumor specimens, including 1,819 SNVs, seven insertions, and 18 deletions (Table S3). Of the SNVs, 163 (9%) were at adjacent genomic positions (DNVs, di‐nucleotide substitutions), including 141 (87%) CC > TT substitutions, a feature of UV‐induced mutagenesis (Rastogi, Richa, Kumar, Tyagi, & Sinha, 2010). Of all mutations, 1,774 (96%) were found in all lesions (*trunk* mutations), suggesting limited mutational heterogeneity between specimens. Trunk mutations comprised *KIT* L576P and *CTNNB1* S33Y. Only 3.8% of the mutations were heterogeneously present between the samples (*branch* and *private*, or *non‐trunk*, Figure 4b). Among these, *CTNNB1* P492S was identified exclusively in PT3, PT5, IT3, and IT4, independently confirming the targeted sequencing data (Figure 4a). Of the 60 genes carrying the non‐trunk mutations, only four belong to Cancer Gene Census (accessed June 18, 2019); *COL3A1*, *CTNNB1*, *FOXO3,* and *SRC*, indicating that the majority of the non‐trunk mutations are passenger mutations. Using the mutation data, we constructed a phylogenetic tree illustrating the evolutionary trajectory of the tumor (Figure 4c). Such analysis did not indicate that primary tumor specimens evolved earlier but rather showed limited dissimilarity between all specimens. There was no difference in the proportion of heterogeneous mutations, that would indicate enrichment for heterogeneity, between PT and IT specimens (*p* = .919).

We then explored the mutational signatures previously described by Alexandrov et al. (2013). All samples displayed predominant UV‐induced DNA damage signature (Figure S6a,b). Interestingly, the distribution of substitution types was significantly different between trunk and non‐trunk mutations (*p* = 3.1 × 10^–6^, Fisher's exact test), with only 57% of non‐trunk SNVs attributable to the UV signature, as compared to 80% among trunk SNVs (*p* = 1 × 10^–4^, Fisher's exact test, Figure 4d).

### 3.5 Copy number heterogeneity in CSD^high^ melanoma

**Figure 5:**
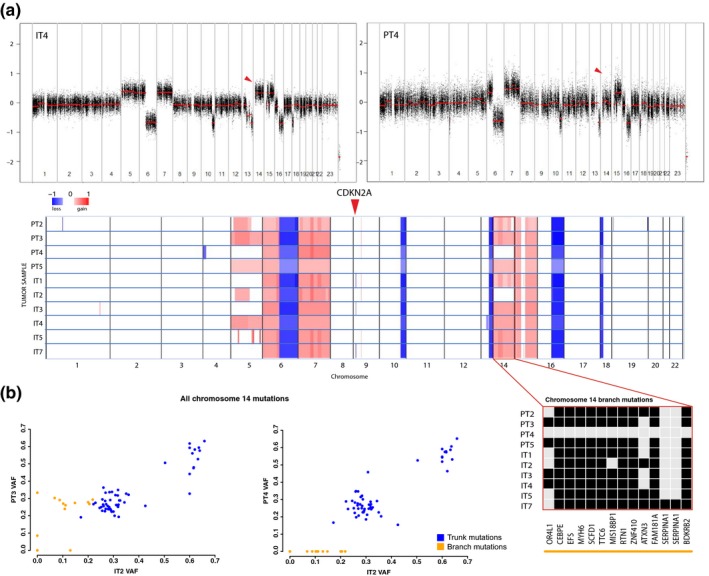
DNA copy number profiles in the CSD^high^ melanoma patient. a, Global copy number profiles of the primary tumor regions and in‐transit metastases, with example profiles from IT4 and PT4 shown on top. Red corresponds to gain and blue to loss. Red arrows indicate loss of CDKN2A on chr 9 and LOH on chr 14. b, Mutations on chr 14. Left panel: VAF of chr 14 branch mutations (in yellow) is lower in IT2 than in PT3, while VAF of trunk mutations (in blue) is comparable between the samples. Middle panel: VAF of chr 14 branch mutations is lower than that of trunk mutations in IT2. Right panel: Zoom‐in on chr 14 copy number with branch mutations depicted. The heterogeneity in copy number level co‐occurs with absence of mutations in PT4

Next, we used WES data for DNA copy number analysis. We found similar aberration profiles, with gains at chr 6p, 7, 15 and losses at 6q, 10q, 13q, 16q, 18p common to all samples (Figure 5a). However, we observed multiple differences. In particular, *CDKN2A* was lost exclusively in IT1, IT3 and IT7. In addition, the ubiquitous copy number gain on chr 14 was absent from PT4 and IT2 (Figure 5a). In PT4, this was reflected in loss of 14 out of 69 mutations on chr 14 (Figure 5b), resulting in its separation from the rest of the samples in the phylogenetic tree (Figure 4c), most probably due to LOH at this region in PT4. In IT2, these mutations, albeit present, show a lower variant allele frequency (VAF, median 10%) than trunk mutations on chr 14 (median 27%; Figure 5b). This may be explained by mixture of clones with and without LOH at chr 14 in IT2. Thus, copy number heterogeneity may partly cause mutational heterogeneity in melanoma tumors.

## 4 DISCUSSION

In this study, we investigated molecular alterations in in situ and invasive CSD^high^ melanoma. We found that CSD^high^ lesions harbored more mutations than CSD^low^ lesions, as previously reported (Berger et al., 2012; Eroglu et al., 2018; Shain, Garrido, et al., 2015). Moreover, our data support earlier findings of increased frequency of *NF1* (Krauthammer et al., 2015) and *BRAF* V600K (Menzies et al., 2012; Stadelmeyer et al., 2014 *)* mutations in CSD^high^ lesions, findings that can have major implications for treatment. For instance, *BRAF* V600K mutant tumors have inferior response to BRAFi as compared to V600E mutant tumors, but respond better to immune checkpoint blockade (Pires da Silva et al., 2019). Importantly, using ultra‐deep sequencing for increased sensitivity in mutation detection in samples with high normal tissue admixture, we were not able to discern differences in mutation frequency of any main melanoma gene between in situ and invasive CSD^high^ lesions. This indicates that CSD^high^ lesions acquire a high number of oncogenic mutations at a very early stage and may not require additional mutations to progress to the invasive phase. Instead, other factors, such as DNA copy number or epigenetic alterations and the host immune system, may be crucial for CSD^high^ in situ lesions to become invasive. However, it should be considered that normal skin epithelium within the CSD^high^ melanoma may harbor an increased mutational load due to the heavy UVR exposure as compared to CSD^low^ melanoma and may thus contribute to the high mutational load of the CSD^high^ in situ lesions. In addition, the CSD^high^ in situ lesions might harbor passenger mutations in the assayed cancer genes, and thus, the elevated mutational load of such lesions may not necessarily reflect an elevated malignant capacity as compared to the CSD^low^ in situ lesions. Finally, in situ CSD^high^ lesions mainly comprised LM, while invasive CSD^high^ lesions were enriched in the SSM subtype (Table 1). Thus, biological differences between these groups may exist and may have contributed to the mutational findings. Nevertheless, in CSD^low^ melanoma, we find an accumulation of somatic mutations from the in situ to the invasive phase, in line with previous reports (Shain, Yeh, et al., 2015). Taken together, our data suggest that tumor progression takes different genetic routes in CSD^high^ and CSD^low^ melanomas.

Intra‐tumor heterogeneity in advanced melanoma is generally not as extensive as in other cancers (Harbst et al., 2016; McGranahan et al., 2015). Herein, we analyzed a CSD^high^ melanoma patient with a synchronous primary and several secondary satellite and in‐transit tumors. From this case, five primary tumor (PT) regions and seven in‐transit (IT) metastases were analyzed. This case harbored several histopathological features characteristic of CSD^high^ melanoma, including high levels of pigmentation, marked solar elastosis, and spindle‐shaped melanoma cells (Smoller, 2006). IT samples exhibited variation in immune cell infiltration and hyperpigmentation patterns that may be explained by biological factors as well as sampling. Although it is always attempted to collect as pure tumor tissue as possible, it is inevitable that normal tissue is present in the sample. In particular, RNA‐seq‐based transcriptional analysis revealed overexpression of immune related genes and decreased expression of immune exclusion genes in PT5 and IT6. However, the overall global transcriptional patterns were highly similar between all specimens, with no differences between PT and IT regions. Further, this similarity was evident also at the mutational level, with the majority of the mutations (>95%) shared by all tumor samples (trunk mutations). While such high similarity at gene expression, mutation and DNA copy number levels may suggest a single lesion, there were no signs of regression. However, such similarity may be in line with the macroscopic appearance; in particular, the symmetric distribution of the metastases around the primary lesion may indicate similar growth kinetics and the same clonal precursor. Trunk mutations included driver mutations in *KIT* (L576P) and *CTNNB1* (S33Y). Of interest, a *CTNNB1* mutation at the same residue (S33C) has been reported to confer resistance to the KIT inhibitor imatinib in a patient with *KIT* L576P mutant melanoma (Cho et al., 2017) and therefore has direct clinical value. Intriguingly, a second *CTNNB1* (P492S) mutation was present only in PT3, PT5, IT3, and IT4. This mutation has not been reported in COSMIC or TCGA (accessed April 18, 2019), and we previously identified heterogeneous *CTNNB1* mutations in multiple metastases following a single primary melanoma (Harbst et al., 2014), indicating that melanomas may harbor passenger *CTNNB1* mutations. However, since these regions are all located in the anatomic vicinity of each other, the accumulation of both mutations in this tumor suppressor gene may have been advantageous for the tumor progression. As expected, the somatic mutations were dominated by the UV mutation signature; however, confirming our previous data, we observed a decreased fraction of UV associated mutations among the non‐trunk mutations (Harbst et al., 2016). Moreover, we observed heterogeneous copy number loss affecting *CDKN2A*, previously associated with melanoma progression (Shain, Garrido, et al., 2015). Our findings indicate that loss of this driver may be heterogeneous in melanoma. Analyses of other multiple metastatic cases of CSD^high^ melanoma are needed in order to conclude on the extent of ITH.

In conclusion, through analysis of a cohort of primary invasive and in situ melanoma, we uncovered mutations in the main melanoma genes in in situ and invasive CSD^high^ melanomas at comparable frequency, indicating that genetic mutations are not the determinants of why only some CSD^high^ in situ lesions progress to invasive melanoma. Additionally, our findings reveal limited molecular diversity within the primary tumor and in‐transit metastasis in a CSD^high^ melanoma case. Our results expand our understanding of CSD^high^ tumor development and progression.

## CONFLICT OF INTERESTS

The authors declare no competing financial interests.
