# Supplementary material for: Tumor genetic heterogeneity analysis of chronic sun‐damaged melanoma
Source: Pigment Cell Melanoma Res. 2019 Dec 23;33(3):480–9. doi: 10.1111/pcmr.12851 (PMC7217060; doi:10.1111/pcmr.12851)
Supplement: Supplementary file 4 [file PCMR-33-480-s004.docx]

**Appendix S1**

**Tumor genetic heterogeneity analysis of chronic sun-damaged melanomas**

Adriana Sanna, Katja Harbst, Iva Johansson, Gustav Christensen, Martin Lauss, Shamik Mitra, Frida Rosengren, Jari Häkkinen, Johan Vallon-Christersson, Håkan Olsson, Åsa Ingvar, Karolin Isaksson, Christian Ingvar, Kari Nielsen, Göran Jönsson

**Supplementary Methods and Figures**

**Supplementary Methods**

**Exome sequencing**. WES data analysis including alignment, post-alignment processing, and variant calling was performed using SAREK pipeline version 2.0.0 (Garcia et al., 2018). In particular, reads were mapped with bwa mem and duplicate fragments were marked using Picard MarkDuplicates. Base Quality Score Recalibration was performed using GATK. Targeted sequencing metrics were derived using Picard CollectHsMetrics. For somatic variant calling, we used 2 algorithms: VarScan v2.4.2 (not part of SAREK) and MuTect2 (part of SAREK). VarScan call sets were further filtered using bam-readcount, VarScan processSomatic and VarScan fpfilter as recommended by VarScan developers, and annotated using Annovar (Wang, Li, & Hakonarson, 2010). Only mutations in the coding sequence of the genes were retained (*i.e.* exonic and splicing). The ubiquitous (trunk) mutations identified by VarScan in all tumor specimens thus constitute the core of the data set. For mutations identified by VarScan in only a proportion of samples (non-ubiquitous mutations) we further looked in MuTect2 call sets to see whether absence of such mutations was due to their variant allele frequency (VAF) <10%. We thus “rescued” mutations using an alternative variant caller. Non-ubiquitous mutations at sites lacking sequence coverage were excluded from the data set. We observed an increased proportion of low variant allele frequency mutations in the private sector as compared to the mutations found in more than 1 sample (Supplementary Figure 1). Since these low VAF private mutations also displayed a different signature composition (i.e., increased proportion of C>A/G>T and decreased proportion of C>T/G>A), pointing to their potential technical artefactual nature, we decided to exclude private mutations with VAF<20% from the analysis. Mutational signatures were obtained with the R package deconstructSigs using signatures.cosmic as input matrix (Rosenthal, McGranahan, Herrero, Taylor, & Swanton, 2016). Copy number analysis, using Contra 2.03 (Li et al., 2012), and phylogenetic analysis were performed as previously described (Lauss et al., 2017). Two samples were removed from the data set due to low overall VAF and compressed copy number profiles owing to dense tumor microenvironment: PT1 and IT6. Mutation data are available in Table S3. **RNA sequencing**. RNA-seq data were analyzed as previously described to obtain FPKM values (Harbst et al., 2016). Then, protein-coding genes as defined by HGNC were retained. To be able to determine whether a gene has relatively low/high expression, we used the TCGA RNAseq data (Cancer Genome Atlas, 2015) as a reference set. We applied the quantile distribution of the quantile-normalized and log-transformed TCGA to each sample of the present data, and centered each gene by subtracting the median gene value of the TCGA data. PT1 exhibited a profile highly similar to the adjacent normal skin sample and was therefore excluded from subsequent analyses. In the supervised analysis of the gene expression data, genes with a standard deviation > 0.3 (6699 genes) were tested for differential expression between PT vs IT group using t-test, and false discovery rate (FDR) using Benjamini-Hochberg correction was reported. Genes with log_2_ fold change above 1 (gene list “Up in IT”) and below -1 (gene list “Up in PT”) between the average of all IT specimens and that of all PT specimens were submitted to GO term analysis using DAVID (Huang da, Sherman, & Lempicki, 2009a, 2009b). Raw sequencing data can be provided upon request.

**Supplementary References**

Cancer Genome Atlas, Network. (2015). Genomic Classification of Cutaneous Melanoma. *Cell, 161*(7), 1681-1696. doi:10.1016/j.cell.2015.05.044

Cirenajwis, H., Lauss, M., Ekedahl, H., Torngren, T., Kvist, A., Saal, L. H., . . . Jonsson, G. (2017). NF1-mutated melanoma tumors harbor distinct clinical and biological characteristics. *Mol Oncol, 11*(4), 438-451. doi:10.1002/1878-0261.12050

Garcia, Maxime, Juhos, Szilveszter, Larsson, Malin, Olason, Pall I., Martin, Marcel, Eisfeldt, Jesper, . . . Käller, Max. (2018). Sarek: A portable workflow for whole-genome sequencing analysis of germline and somatic variants. *bioRxiv*, 316976. doi:10.1101/316976

Harbst, K., Lauss, M., Cirenajwis, H., Isaksson, K., Rosengren, F., Torngren, T., . . . Jonsson, G. (2016). Multiregion Whole-Exome Sequencing Uncovers the Genetic Evolution and Mutational Heterogeneity of Early-Stage Metastatic Melanoma. *Cancer Res, 76*(16), 4765-4774. doi:10.1158/0008-5472.CAN-15-3476

Huang da, W., Sherman, B. T., & Lempicki, R. A. (2009a). Bioinformatics enrichment tools: paths toward the comprehensive functional analysis of large gene lists. *Nucleic Acids Res, 37*(1), 1-13. doi:10.1093/nar/gkn923

Huang da, W., Sherman, B. T., & Lempicki, R. A. (2009b). Systematic and integrative analysis of large gene lists using DAVID bioinformatics resources. *Nat Protoc, 4*(1), 44-57. doi:10.1038/nprot.2008.211

Lauss, M., Donia, M., Harbst, K., Andersen, R., Mitra, S., Rosengren, F., . . . Jonsson, G. (2017). Mutational and putative neoantigen load predict clinical benefit of adoptive T cell therapy in melanoma. *Nat Commun, 8*(1), 1738. doi:10.1038/s41467-017-01460-0

Li, J., Lupat, R., Amarasinghe, K. C., Thompson, E. R., Doyle, M. A., Ryland, G. L., . . . Gorringe, K. L. (2012). CONTRA: copy number analysis for targeted resequencing. *Bioinformatics, 28*(10), 1307-1313. doi:10.1093/bioinformatics/bts146

Rosenthal, R., McGranahan, N., Herrero, J., Taylor, B. S., & Swanton, C. (2016). DeconstructSigs: delineating mutational processes in single tumors distinguishes DNA repair deficiencies and patterns of carcinoma evolution. *Genome Biol, 17*, 31. doi:10.1186/s13059-016-0893-4

Wang, K., Li, M., & Hakonarson, H. (2010). ANNOVAR: functional annotation of genetic variants from high-throughput sequencing data. *Nucleic Acids Res, 38*(16), e164. doi:10.1093/nar/gkq603

**Supplementary Figure 1.**

**Supplementary Figure 2**.

A.

All lesions

B.

CSD^high^ CSD^low^

**Supplementary Figure 3**.

A. B.

**Supplementary Figure 4**.

**
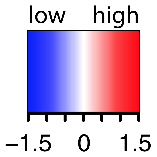
**

**Supplementary Figure 5.**

A. ****

B.****

**Supplementary Figure 6**.
